# Novel Mechanistic Insights into Viral Modulation of Immune Receptor Signaling

**DOI:** 10.1371/journal.ppat.1000404

**Published:** 2009-07-31

**Authors:** Alexander B. Sigalov

**Affiliations:** Department of Pathology, University of Massachusetts Medical School, Worcester, Massachusetts, United States of America; The Scripps Research Institute, United States of America

To successfully infect, replicate, and persist in the host, viruses have evolved numerous strategies to take control of multiple cellular processes, including those that target transmembrane (TM) signal transduction mediated by immune receptors. Despite tremendous advancement in recent years, the exact molecular mechanisms underlying these critical points in viral pathogenesis remain unknown. In this Opinion, based on a novel model of immune signaling, the Signaling Chain HOmoOLigomerization (SCHOOL) model, I suggest specific mechanisms used by different viruses such as human immunodeficiency virus (HIV), cytomegalovirus (CMV), severe acute respiratory syndrome coronavirus (SARS-CoV), herpesvirus saimiri (HVS), human herpesvirus 6 (HHV-6), etc., to modulate the host immune response mediated by members of the family of multichain immune recognition receptors (MIRRs). I also demonstrate how the SCHOOL model, together with the lessons learned from viral pathogenesis, can be used practically for rational drug design and the development of new therapies for immune disorders.

In MIRRs, the recognition domains and signaling sequences containing immunoreceptor tyrosine-based activation motifs (ITAMs) are located on separate subunits bound together by noncovalent TM interactions (Figure 1A) [1]–[3]. Based on a novel biophysical phenomenon, the homointeractions of intrinsically disordered proteins [4],[5], the SCHOOL model of MIRR signaling [3], [6]–[8] uncovers the molecular mechanisms by which clustering of the extracellular recognition domains leads to receptor triggering. The model suggests that MIRR engagement leads to receptor oligomerization coupled with a multi-step structural reorganization driven by the homooligomerization of signaling subunits (Figure 1B). Importantly, this model is based on specific protein–protein interactions—biochemical processes that can be influenced and controlled, providing a promising drug design approach [9].

**Figure 1:**
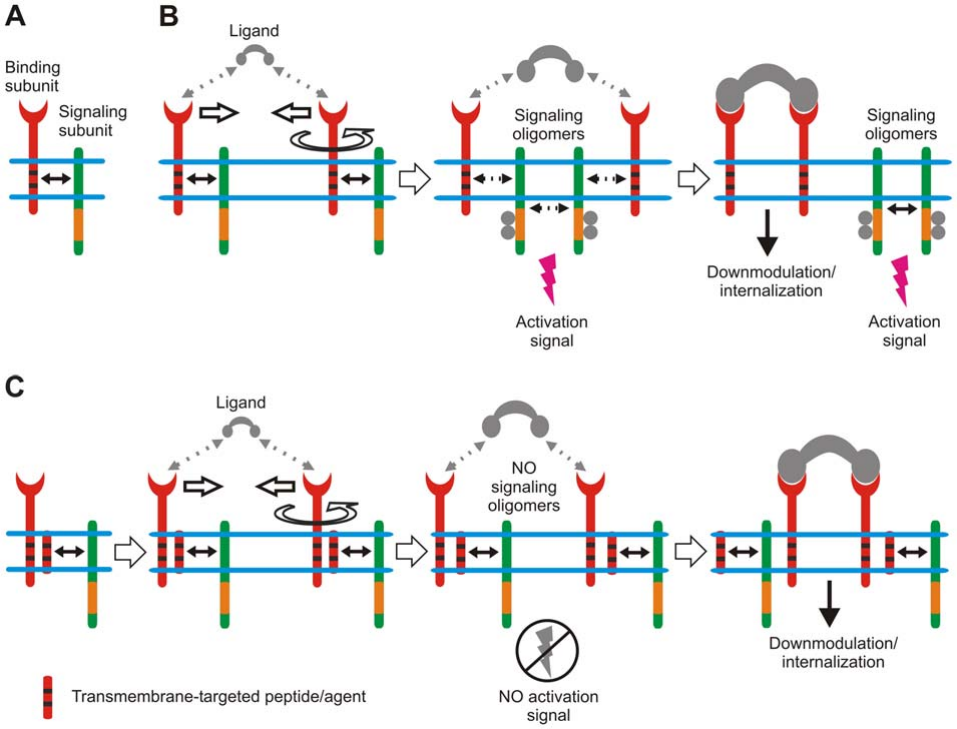
Novel model of immune signaling reveals a new target and tools for immunomodulatory intervention. (A) Multichain immune recognition receptor (MIRR) assembly. Binding and signaling subunits are shown in red and green, respectively. ITAMs are shown as orange rectangles. Transmembrane interactions between MIRR ligand-binding and signaling components (shown by solid arrow) play a key role in receptor assembly and integrity on resting cells. (B) The signaling chain homooligomerization (SCHOOL) model, which proposes that the homooligomerization of signaling subunits plays a central role in triggering MIRRs. Small solid black arrows indicate specific intersubunit hetero- and homointeractions between transmembrane and cytoplasmic domains, respectively. Circular arrow indicates ligand-induced receptor reorientation. All interchain interactions in a dimeric intermediate are shown by dotted black arrows reflecting their transition state. Phosphate groups are shown as dark circles. (C) Molecular mechanisms underlying proposed immunomodulatory intervention by transmembrane-targeted agents. Specific blockade of transmembrane interactions between MIRR recognition and signaling subunits results in “predissociation” of the receptor complex, thus preventing formation of signaling oligomers and inhibiting ligand-dependent immune cell activation. In contrast, stimulation of these predissociated MIRRs with cross-linking antibodies to signaling subunits should still lead to receptor triggering and cell activation (not shown).

Within the model, specific blockade or disruption of TM interactions causes a physical and functional disconnection of the MIRR subunits (Figure 1C) [6],[7],[10],[11]. Antigen stimulation of these “predissociated” receptors leads to reorientation and clustering of the recognition but not signaling subunits. As a result, signaling oligomers are not formed, ITAM Tyr residues do not become phosphorylated, and the signaling cascade is not initiated (Figure 1C). In contrast, this “predissociation” does not prevent the formation of signaling oligomers when signaling subunits are clustered by specific antibodies that trigger cell activation (not illustrated).

Predicted and molecularly explained by the SCHOOL model [7], [10]–[13], manipulation of MIRR signaling is performed by numerous unrelated viruses throughout their life cycle. In this context, the ability viruses have developed over centuries of evolution [13],[14] to modulate T cell receptor (TCR) signaling plays a crucial role in viral pathogenesis. For T lymphotropic viruses, the virus may inhibit TCR signaling to disarm the receptor and successfully enter the cell [7],[10],[11],[13],[14]. A similar strategy can be used by the virus to persist in the cell until it reactivates and produces infectious particles. For other viruses, modulation of TCR signaling can be used to inhibit the T cell response to infected cells [7],[11],[13],[14]. Structurally, TCR is a member of the MIRR family and has the α and β antigen-binding subunits that are bound by electrostatic TM interactions with three signaling homo- and heterodimers: ζζ, CD3εδ, and CD3εγ (Figure 2A) [2],[15]. As suggested by the SCHOOL model [7],[10],[11],[13], these interactions are not only promising therapeutic targets, but also represent an important point of viral attack.

**Figure 2:**
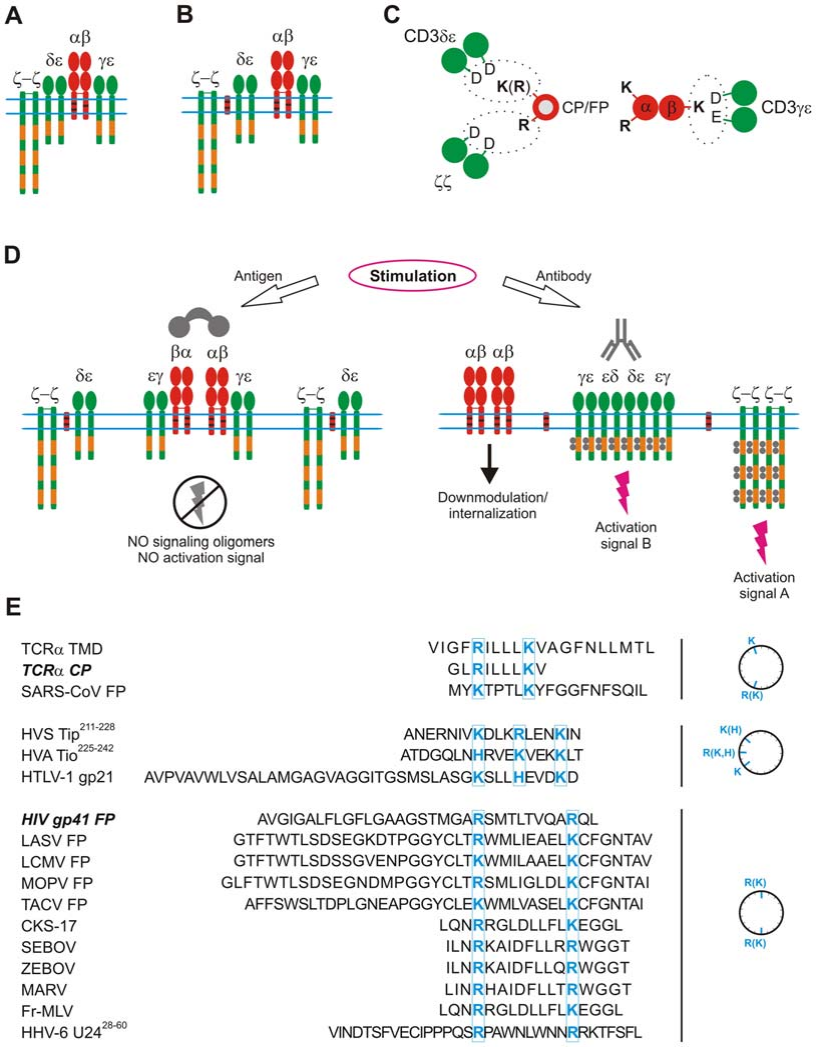
Molecular mechanisms suggested by the SCHOOL model to be used by diverse viruses to modulate TCR signaling. (A) TCR assembly. The α and β binding subunits are shown in red. The CD3ε, CD3δ, CD3γ, and ζ signaling subunits are shown in green. ITAMs are shown as orange rectangles. Within the SCHOOL model, transmembrane-targeted agents such as TCR core peptide (CP) or HIV gp41 fusion peptide (FP) disrupt the transmembrane electrostatic interactions between the ligand-binding TCRα chain and CD3δε and ζζ by competing with the TCRα chain for binding to CD3δε and ζζ (B). This results in functional disconnection of the relevant signaling subunits ([C], shown as a simplified axial view) and prevents formation of signaling oligomers upon antigen but not antibody stimulation, thus inhibiting antigen-mediated but not anti-CD3-mediated TCR triggering and cell activation (D). Primary sequence analysis of proven and predicted immunomodulatory sequences of viral fusion protein regions and other domains shows a similarity in the charge distribution pattern with two essential positively charged residues (shown in blue) spaced apart by three to four or seven to eight amino acids (E), suggesting a similarity of mechanisms used by diverse viruses in their pathogenesis to modulate the host immune response. Note: Although the three-dimensional structures of the analyzed sequences within the cell membrane are not known, it might be assumed that these sequences may adopt a helical conformation upon membrane binding. Thus, helical wheel projections are used for illustrative purposes only; the suggested mode of action does not depend on a particular secondary structure of the sequences. Abbreviations: CKS-17, a synthetic retroviral envelope heptadecapeptide; Fr-MLV, Friend murine leukemia virus; gp, glycoprotein; HHV-6 U24, human herpesvirus 6 U24 protein; HTLV-1, human T lymphotropic virus type 1; HVA, herpesvirus ateles; HVS, herpesvirus saimiri; ITAM, immunoreceptor tyrosine-based activation motif; LASV, Lassa virus; LCMV, lymphocytic choriomeningitis virus; MARV, Marburg virus; MOPV, Mopeia virus; SARS-CoV, severe acute respiratory syndrome coronavirus; SEBOV, Sudan Ebola virus; TACV, Tacaribe virus; Tip, tyrosine kinase interacting protein; Tio, two-in-one protein; TMD, transmembrane domain; ZEBOV, Zaire Ebola virus.

TM peptides capable of inhibiting TCR-mediated cell activation were first reported in 1997 [16]. The vast majority of findings were reported for the TCR core peptide (CP), a synthetic peptide corresponding to the sequence of the TCRα TM domain (TMD) and known to interact with the TMDs of CD3δε and ζ [2],[15]. Interestingly, T cell activation via anti-CD3 antibodies is not affected by this peptide (Table 1). As shown, TCR CP might be a proper treatment for human T cell-mediated dermatoses substituting for corticosteroids [17],[18]. However, despite extensive studies [2], [17]–[28], the mode of action of this clinically relevant peptide was not explained until 2004 when the SCHOOL model was first introduced [6].

**Table 1:** Similarities in Characteristics and Immunomodulatory Activities of the T Cell Receptor Core Peptide and HIV-1 gp41 Fusion Peptide.

Characteristics/Activation Model	CP [17], [24]–[26]	FP [29]
Colocalization with TCR	+	+
Coprecipitation with TCR	+	+
Immunosuppressive activity in vivo	+	+
Inhibition of in vitro activation:		
Antigen	+	+
Anti-TCRβ antibody	—	ND
Anti-CD3 antibody	—	—
PMA/ionomycin	—	—

TCR, T cell receptor; CP, core peptide; FP, fusion peptide; ND, not determined; PMA, phorbol 12-myristate 13-acetate.

Recently, inhibition of T cell activation has been reported for the fusion peptide (FP) found in the N terminus of the HIV envelope glycoprotein 41 (gp41) [29],[30]. These data are the first to demonstrate that not only does FP function to fuse the virion with the host cell membrane [31],[32], but it also has immunomodulatory activity. The peptide inhibits antigen- but not anti-CD3-stimulated T cell activation in vitro and has immunosuppressive activity in mice [29] (Table 1). Similar to TCR CP [18],[20],[22],[23], HIV FP has been suggested for the treatment of T cell-mediated pathologies [29]. However, the mode of action of this peptide remained unexplained until 2006 when the SCHOOL model was first applied to this area [7].

Considering the similarity between FP and CP in patterns of immunomodulatory activity (Table 1) and in their having two electropositive residues in their primary sequences, the SCHOOL model reasonably suggests a similar mode of action for these peptides (Figure 2) [3], [6], [7], [10]–[12]. Briefly, CP and FP compete with TCRα for binding to CD3δε and ζζ, resulting in functional disconnection of these subunits (Figure 2C). This prevents formation of CD3δε and ζ signaling oligomers and thus inhibits T cell activation upon stimulation with antigen but not anti-CD3 antibodies (Figure 2D), thereby suggesting a molecular explanation for the use of OKT3 antibodies in HIV therapy to augment immune activation [33]. Interestingly, the SCHOOL mechanism is the only one consistent with all of the experimental data on the immunomodulatory action of HIV FP and TCR CP reported so far [16], [21], [24]–[26], [28]–[30].

Charge distribution patterns for fusion protein regions are surprisingly conserved in many unrelated viruses and show similarities to those for TCR CP and HIV FP (Figure 2E). Thus, it is highly probable that these proteins would also target the TCR TM interactions using the SCHOOL mechanism. Exploratory sequence investigation of FPs from SARS-CoV, Lassa virus (LASV), lymphocytic choriomeningitis virus (LCMV), Mopeia virus (MOPV), and Tacaribe virus (TACV) reveals a close similarity in the positioning of the electropositive residues (Figure 2E). Intriguingly, analysis of other unrelated viruses has yielded similar correlations in primary structure and function. Earlier studies have reported an inhibitory effect on lymphocyte proliferation by CKS-17 peptide, a synthetic 17-mer peptide with sequence corresponding to a highly conserved region of TM proteins of human and animal retroviruses, including the TM protein gp21 of human T lymphotropic virus type 1 (HTLV-1) [34]–[36]. Interestingly, peptides corresponding to regions of HIV TM protein gp41 homologous to the highly conserved and immunosuppressive sequence contained within the TM proteins p15E and gp21 of animal and human retroviruses, respectively, also have been reported to inhibit lymphoproliferation [35],[36]. Recently, filoviral 17-mer peptides corresponding to a 17–amino acid domain in filoviral glycoproteins that resembles an immunosuppressive motif in retroviral envelope proteins have been demonstrated to inhibit TCR-mediated cell activation [37]. In all peptides, a striking similarity is observed in the charge distribution patterns with the positioning of the essential positively charged residues almost identical to that for the HIV gp41 FP (Figure 2E), suggesting again a similarity in their mode of action. This clearly demonstrates that different viruses have adopted similar mechanisms of specifically targeting TCR, disrupting receptor architecture, and suppressing the immune system. Importantly, by virtue of the acquired insight into this conserved structural motif, expanded predictions, hypotheses, and conclusions can be derived to being answering the question of whether shared TCR-targeted strategies represent a conserved function or a convergent tactic of divergent viruses.

The generality of the SCHOOL model suggests that TM interactions of other MIRRs can also represent a point of viral attack. As reported [38], the recognition of the human CMV tegument protein pp65 by NKp30, the natural killer (NK) cell-activating receptor, does not lead to NK cell activation but instead results in a general inhibition mediated by the dissociation of the NKp30-ζ complex and a loss in the ability of cells to kill virus-infected cells. Within the context of the SCHOOL model, pp65 may target the TM interactions between NKp30 and ζ, leading to functional disconnection of ζ in a manner similar to the action of TCR CP and HIV FP (Figure 2).

TM interactions can be targeted not only from outside but also from inside the cell. Recently, it has been shown that the HHV-6 U24 protein downregulates TCR surface expression and that U24-expressing T cells are resistant to activation by antigen-presenting cells [39]. By controlling lymphocyte activation that is often accompanied by herpesvirus reactivation, the virus might prevent its own reactivation and persist in a latent state, which is less prone to immune recognition [39]. In this context, U24 can serve to maintain equilibrium between the virus and its host by keeping HHV-6 titers low enough that they do not cause massive immune activation [39]. TCR downregulation activity also has been reported for the highly conserved membrane-proximal sequence of the tyrosine kinase-interacting protein (Tip) of HVS [40],[41]. Notably, primary sequences of HHV-6 U24^28–60^ and HIV FP exhibit a similar pattern with two Arg residues spaced apart by eight amino acids (Figure 2E). The positioning of the essential electropositive residues is remarkably conserved in HVS Tip^213–228^, the relevant domain of the two-in-one (Tio) protein of herpesvirus ateles (HVA) and HTLV-1 gp21 (Figure 2E). Thus, the SCHOOL mechanisms similar to those applied for TCR CP and HIV gp41 FP (Figure 2) can be used by HHV-6 and other viruses in their arsenal of immune evasion tactics. Importantly, as predicted, the viral agents prevent only antigen- but not antibody-specific, T cell activation (Figure 2D). Indeed, anti-CD3 antibodies activate HHV-6-infected T cells, resulting in a large increase of viral replication [42],[43]. Interestingly, increase of viral replication induced by OKT3-mediated activation of HIV-infected T cells is currently used for purging of the latent HIV-1 reservoirs in vivo [33], thus suggesting a potential generality of the SCHOOL mechanism-based antiviral approaches.

There are several important lessons that we can learn from the molecular mechanisms of viral pathogenesis. First, using modern methodologies [44]–[51], it is possible to design and produce TM agents that are able to modulate the immune response as specifically and effectively as viruses do. Second, as predicted, TCR CP and many different immunomodulatory viral sequences affect similar TCR–TM interactions, suggesting that general principles of designing TM peptides might be readily used at this stage [47],[48]. Third, antibodies to MIRR signaling subunits can be used to modulate the affected immune cell response during viral infection. Fourth, considering our selective ability to functionally disconnect any particular TCR signaling subunits [8],[10],[11],[52], we can use the relevant peptides as a powerful tool to dissect fine mechanisms of viral pathogenesis. Finally, two unrelated viruses, HIV and human CMV, use a similar mode of action to modulate the host immune response mediated by two functionally different MIRRs, TCR and NKp30, thus suggesting that similar general mechanisms can be or are used by other viral and possibly non-viral pathogens.

In conclusion, rather than targeting virus-specific proteins or processes, it would be advantageous to transfer therapeutic strategies that target redundant processes found among a number of viruses. In addition, as demonstrated by the similar function of natural HIV FP and synthetically derived clinically relevant TCR CP, viral immune evasion strategies can be transferred to therapeutic strategies that require similar functionalities. Viruses represent years of evolution and the efficiency and optimization that come along with it. Therefore, viral functions should not only be studied as foreign processes but as efficient strategies that we can use in our own attempts at immune evasion or immunomodulation.

## Sequence Accession Numbers

Accession numbers (UniProtKB/Swiss-Prot knowledgebase, http://www.expasy.org/sprot/) for the viruses discussed in this Opinion are: CMV, P06725; Fr-MLV, P03390; HHV-6, Q69559; HIV-1, P04578; HTLV-1; P03381; HVA, Q9YJQ8; HVS, P22575; LASV, P08669; LCMV, P07399; MARV, P35253; MOPV, P19240; SARS-CoV, P59594; SEBOV, Q66814; TACV, P18141; ZEBOV, Q05320.
